# Longevity

**Published:** 1897-12

**Authors:** 


### Longevity.

It is not an uncommon contention that the stories about persons having passed the century mark are "fairy tales" and that really the limit of human life is under 100 years. But statistics show beyond a doubt that the span of life in many cases is more than a century, and that old age among certain races is nearer ninety than seventy. During the ten years from 1881 to 1890, inclusive, there were in Massachusetts 203 deaths of persons past the age of 100. Of these centenarians 165 were between 100 and 105, thirty-five were between 105 and 110, five were between 110 and 115 and one was 118. Of the 203 there were 153 females and fifty males. In Iowa there are now more than 500 persons beyond the age of ninety. There are twenty-one who are more than 100 years old. A statistician named Haller collected a number of instances of extreme longevity in England. He found 1,000 persons who lived from 100 to 110 years, sixty persons who lived from 110 to 120 years, twenty-nine who lived from 120 to 130 years, fifteen persons from 130 to 140 years, six persons from 144 to 150, and one person who lived to the remarkable age of 169. Henry Jenkins was born in Yorkshire in 1501 and died in 1670. It was proved from the registers of the chancery and other courts that he had appeared in evidence 140 years before his death and had had an oath administered to him. In the office of the King's Remembrancer there appears a record of a deposition in which he appears as a witness at 157.

